# Transcriptomic Analysis of the *Brucella melitensis* Rev.1 Vaccine Strain in an Acidic Environment: Insights Into Virulence Attenuation

**DOI:** 10.3389/fmicb.2019.00250

**Published:** 2019-02-14

**Authors:** Mali Salmon-Divon, Tamar Zahavi, David Kornspan

**Affiliations:** ^1^Genomic Bioinformatics Laboratory, Department of Molecular Biology, Ariel University, Ariel, Israel; ^2^Department of Bacteriology, Kimron Veterinary Institute, Bet Dagan, Israel

**Keywords:** *Brucella melitensis* 16M, *Brucella melitensis* Rev.1, acid stress, attenuation, virulence, transcriptomic analyses, RNA-Seq

## Abstract

The live attenuated *Brucella melitensis* Rev.1 (Elberg-originated) vaccine strain is widely used to control the zoonotic infection brucellosis in small ruminants, but the molecular mechanisms underlying the attenuation of this strain have not been fully characterized. Following their uptake by the host cell, *Brucella* replicate inside a membrane-bound compartment—the *Brucella*-containing vacuole—whose acidification is essential for the survival of the pathogen. Therefore, identifying the genes that contribute to the survival of *Brucella* in acidic environments will greatly assist our understanding of its molecular pathogenic mechanisms and of the attenuated virulence of the Rev.1 strain. Here, we conducted a comprehensive comparative transcriptome analysis of the Rev.1 vaccine strain against the virulent reference strain 16M in cultures grown under either normal or acidic conditions. We found 403 genes that respond differently to acidic conditions in the two strains (FDR < 0.05, fold change ≥ 2). These genes are involved in crucial cellular processes, including metabolic, biosynthetic, and transport processes. Among the highly enriched genes that were downregulated in Rev.1 under acidic conditions were acetyl-CoA synthetase, aldehyde dehydrogenase, cell division proteins, a cold-shock protein, GroEL, and VirB3. The downregulation of these genes may explain the attenuated virulence of Rev.1 and provide new insights into the virulence mechanisms of *Brucella*.

## Introduction

*Brucella* are facultative intracellular bacteria that are responsible for brucellosis—a zoonotic infection that causes abortions and sterility in ruminants, pigs, dogs, and rodents, and a severely debilitating febrile illness in humans (Ko and Splitter, 2003; von Bargen et al., 2012). One factor that crucially contributes to the virulence of *Brucella* is their ability to survive within various host cells, where they are inaccessible to the humoral immune response of the host (Delrue et al., 2004). Following uptake by the host cells, *Brucella* create a unique, highly acidic intracellular niche—the *Brucella*-containing vacuole (BCV)—in which they reside and multiply (Celli, 2006; Starr et al., 2008). The acidification of the BCV is essential for inducing the major *Brucella* virulence determinant, the type-IV secretion system (T4SS; Porte et al., 1999; Boschiroli et al., 2002; Köhler et al., 2002; Ke et al., 2015) which is encoded by the *virB* locus in their chromosomes. As the T4SS system (and, especially, the proteins VirB3–6 and VirB8–11) plays a crucial role in inhibiting the host immune response and in the intracellular survival and replication of *Brucella* within the host cells (Comerci et al., 2001; den Hartigh et al., 2008; Ke et al., 2015; Smith et al., 2016), the ability of *Brucella* to survive within the acidic conditions of the BCV is key to their pathogenesis and can be used to study the underlying mechanisms (Roop et al., 2009).

Porte et al. (1999) reported that the pH in phagosomes containing live Brucella *suis* decreases to 4.0 within 1 h following infection, and that this value persists for at least 5 h. Thus, one can assume the existence of a complex, transcription-level regulation network, which responds to specific cellular signals that enable the bacteria to survive in the acidic BCV environment. Indeed, two recent comparative transcriptome analyses employed RNA-seq to determine the changes in *Brucella* gene expression in cultures containing normal-pH media (namely, pH_7.3_) versus those containing low-pH media (pH_4.4_), thereby revealing novel molecular mechanisms leading to *Brucella* pathogenicity (Liu et al., 2015, 2016). Notably, one gene that was shown to play an important role in the resistance of *Brucella* to low-pH conditions is BMEI1329, which encodes a two-component response regulator gene in the transcriptional regulation pathway of *Brucella melitensis* (Liu et al., 2016).

*Brucella melitensis*, which infects goats and sheep mainly around the Mediterranean and the Persian Gulf, is the most pathogenic *Brucella* species for humans (Poester et al., 2013). Among the brucellosis vaccines used in high-prevalence regions, a widely used one utilizes the live attenuated Rev.1 *B. melitensis* strain (Avila-Calderón et al., 2013). This strain, originally developed from the virulent *B. melitensis* 6056 strain by Elberg and Herzberg in the mid-1950s, successfully protects and reduces abortions in small ruminants (Herzberg and Elberg, 1953; Banai, 2002), but it remains infectious for humans and causes abortions in small ruminants vaccinated during the last trimester of gestation. To improve brucellosis vaccines, we need to better understand the mechanisms underlying the virulence attenuation of the Rev.1 vaccine strain (as compared with that of other, pathogenic strains), but these mechanisms are yet unclear.

In a recent study, we sequenced and annotated the whole genome of the original Elberg *B. melitensis* Rev.1 vaccine strain (passage 101, 1970) and compared it to that of the virulent *B. melitensis* 16M strain (Salmon-Divon et al., 2018a,b). We found that, as compared with 16M, Rev.1 contains non-synonymous and frameshift mutations in important virulence-related genes—including genes involved in lipid metabolism, stress response, regulation, amino acid metabolism, and cell-wall synthesis—which we assumed are related to the attenuated virulence of this strain. In this study, we aimed to extend these findings to elucidate the intracellular survival mechanisms of the virulent 16M strain versus the vaccine Rev.1 strain. To this end, and in light of the importance of the acidic BCV environment for the virulence of *Brucella* species, we employed RNA-seq to comprehensively compare the transcriptome of the Rev.1 and 16M strains, each grown under either low- or normal-pH conditions, under the hypothesis that the gene expression patterns of the two strains will differ between the two conditions. Our analysis revealed several candidate genes that may be related to the attenuated virulence of Rev.1 and may, therefore, facilitate the design of improved brucellosis vaccines.

## Materials and Methods

### Bacteria Strains and Culture Conditions

Bacterial strains used in the present study were *B. melitensis* 16M (INRA *Brucella* Culture Collection)—the commonly used, virulent, wild-type biotype 1 strain—and the original attenuated *B. melitensis* Rev.1 vaccine strain (passage 101, 1970). For comparative assays, both the Rev.1 and 16M strains were cultured for 72 h on tryptic soy agar (TSA) plates at 37°C under 5% CO_2_. The low-pH treatment assay was performed as reported previously (Liu et al., 2016). Briefly, bacteria were grown with shaking for 24 h in 10 ml of a tryptic soy broth (TSB; pH_7.3_) at 37°C, with an initial density of 1 × 10^7^ CFU/ml. The final bacterial densities were adjusted to 5 × 10^8^ CFU/ml (OD_600_ ≅ 0.4) before the low-pH treatment, in which 1 ml of the culture was centrifuged at 7000 ×*g*, resuspended in a pH_4.4_ TSB culture, and incubated for 4 h at 37°C. In the control group, bacteria were cultured in a pH_7.3_ TSB and incubated at 37°C for 4 h. After incubation, cell cultures were collected and centrifuged at 7000 ×*g*, and then the supernatants were removed and an RNA Protect Reagent (Qiagen, Hilden, Germany) was added to the pellets to prevent RNA degradation. Five different biological replicates were used for each strain under each type of condition (total 20 samples). All the work with *Brucella* strains was performed at a biosafety level 3 laboratory in the Kimron Veterinary Institute, Bet Dagan, Israel.

### RNA Isolation

The total RNA of the 16M and Rev.1 strains was isolated using the RNeasy Mini Kit (Qiagen) with a DNase treatment (Qiagen). RNA was eluted from the column using RNase-free water. RNA quality was measured by Bioanalyzer (Agilent, Waldbronn, Germany). Libraries were prepared using the ScriptSeq RNA-Seq Library Preparation Kit (Illumina, Inc., San Diego, CA, United States). Library quantity and pooling were measured by Qubit [dsDNA high sensitivity (HS); Molecular Probes, Inc., Eugene, OR, United States]. The pool was size-selected by using a 4% agarose gel. Library quality was measured by TapeStation (HS; Agilent). For RNA-seq, the NextSeq 500 high output kit V2 was used (Illumina, Inc.). The reads were single end at the length of 75 bp (∼10 million reads per sample). Sample denaturation and loading were conducted according to the manufacturer’s instructions. Library preparation and RNA-seq were conducted at the Center for Genomic Technologies at the Hebrew University of Jerusalem, Jerusalem, Israel.

### Reverse Transcriptase PCR (RT-PCR)

To confirm the RNA-seq results, five upregulated or downregulated genes from the RNA-seq analysis were selected and a RT-PCR was used to confirm the expression changes of these genes in both strains (16M and Rev.1) and conditions (low- and normal-pH). PCR primers were designed using Primer-BLAST (Ye et al., 2012) and are listed in Supplementary Table S1. Complementary DNA (cDNA) was obtained by a reverse transcription of 850 ng total RNA at a final reaction volume of 20 μl, containing 4 μl qScript Reaction Mix, and 1 μl qScript Reverse Transcriptase (Quantabio, Beverly, MA, United States). Quantitative RT-PCR assays were purchased from Biosearch Technologies (Petaluma, CA, United States) and used according to the manufacturer’s instructions. PCR reactions were conducted in a final reaction volume of 10 μl containing 20 ng of cDNA template, 5 μl of PerfeCTa SYBR Green FastMix, ROX (Quantabio), and 1 μl of primer mix. All reactions were run in triplicate and the reference gene 16S rRNA was amplified in a parallel reaction for normalization.

### RNA-Seq Analysis

Following quality control with FastQC^1^, the reads were processed to trim adaptors and low-quality bases by using Trim Galore software^2^. The EDGE-pro v1.3.1 software (Magoc et al., 2013) was used with the default parameters to map reads to the *B.* *melitensis* 16M reference genome (GCF_000007125.1), filter out multi-aligned reads, and estimate the expression levels of each gene. To convert the EDGE-pro output to a count-table format, the “edgeToDeseq.perl” script (provided with the software) was used. Normalization and differential gene expression analysis were conducted with the edgeR (Robinson et al., 2010) and Limma R packages (Smyth, 2005), using as input the count table generated by EDGE-pro. Briefly, genes that did not show more than 1 count per million (CPM) mapped reads in at least three samples were filtered out. Then, a TMM normalization (Robinson and Oshlack, 2010) was applied, followed by voom transformation (Law et al., 2014). Linear models to assess differential gene expression were generated by fitting a model with a coefficient for all factor combinations (strain and low-pH treatment) and then extracting the comparisons of interest, which also included the interaction between strain and treatment effects. The aim of adding the interaction term in this experimental setup was to detect genes that respond differently to pH treatment in Rev.1 compared to 16M; we named these genes “interaction genes.” Only genes that demonstrated a fold change ≥ 2 and an FDR ≤ 0.05 were considered significant. Sequencing reads from this study were deposited in the NCBI SRA repository under the accession number PRJNA498082. The significantly upregulated or downregulated genes were subjected to a gene ontology enrichment analysis using ClusterProfiler (Yu et al., 2012) with a cutoff of FDR < 0.05. To perform the gene ontology analysis, we first generated a database annotation package for *B. melitensis* 16M using the “makeOrgPackage” command from the AnnotationForge R package (Carlson, 2018). As input, we used the GO annotation, downloaded from QuickGO (Binns et al., 2009). Additional comparisons of the biological processes were performed with the Comparative GO web server (Fruzangohar et al., 2013) using all the upregulated and downregulated genes. Multidimensional scaling analysis (MDS) was conducted using the “plotMDS” command within the edgeR (Robinson et al., 2010) package. A heatmap of the 403 genes that respond differently to acidic conditions in the two strains was generated using the “heatmap3” R package (Zhao et al., 2014), employing 1-Pearson correlation as the distance measure and “complete” as the linkage method. Genes were categorized into five clusters based on the generated dendrogram, and genes within each cluster were characterized based on Clusters of Orthologous Groups (COGs) annotations. For this purpose, protein sequences of the clustered genes were searched against a local COG BLAST database, which was downloaded from NCBI using the reverse position-specific BLAST (RPS-BLAST) tool (Marchler-Bauer et al., 2013). The expectation value (E) threshold was set to 0.01 and the BLAST output was parsed using an updated version of the cdd2cog.pl script^3^ to obtain the assignment statistics of the COGs. The number of genes within each heatmap cluster belonging to each COG assignment was calculated, and the ontologies with the highest number of genes were indicated in the heatmap.

### Cell Infection Test

JEG-3 (ATCC^®^ HTB-36^TM^) human trophoblasts were grown in Eagle’s Minimum Essential Medium (EMEM; ATCC^®^ 30-2003^TM^) with 10% fetal bovine serum. For intracellular replication experiments, 2 × 10^5^ cells were seeded in a 24-well plate and cultured overnight at 37°C under 5% CO_2_. Monolayers of cells were infected with the 16M or Rev.1 strains at a multiplicity of infection (MOI) of 500 (100 μl of bacterial suspension per well). To synchronize the infection, the infected plates were centrifuged at 400 g for 5 min at room temperature, followed by a 75 min incubation at 37 °C in an atmosphere containing 5% CO_2_. The cells were then washed three times with PBS and re-incubated for another 60 min in a medium containing 50 μg/ml gentamicin to eliminate extracellular bacteria, after which the number of internalized bacteria was measured (time zero of the culture). To assess the intracellular bacterial growth, the concentration of gentamicin was reduced to 5 μg/ml. To monitor the intracellular survival of the bacteria at various times post-infection, the infected cells were lysed for 10 min with 0.1% Triton X-100 in water and serial dilutions of the lysates were plated on TSA plates to enumerate the colony-forming units. Three identical wells were evaluated at each time for each strain. Experiments were repeated three times, independently.

## Results

**FIGURE 1:**
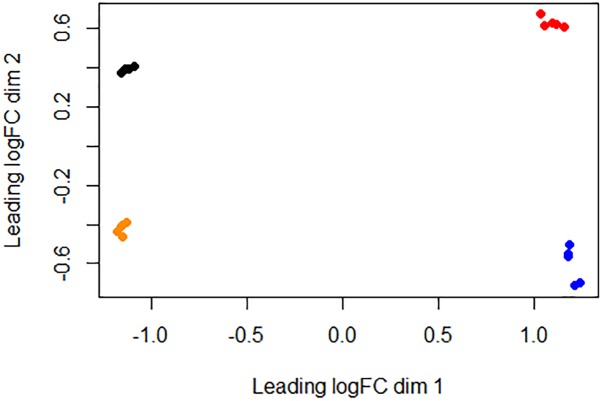
Similarities between bacterial samples visualized using an MDS analysis. Relative distances between bacterial samples were projected onto a two-dimensional space using the “plotMDS” command implemented at the Limma R package (Smyth, 2005). Black, 16M normal-pH group samples; red, 16M low-pH group samples; orange, Rev.1 normal-pH group samples; blue, Rev.1 low-pH group samples.

We used RNA-seq to conduct a comprehensive comparative transcriptomic analysis of the gene expression profiles of the Rev.1 (vaccine) and 16M (virulent) *B. melitensis* strains, grown either under low-pH conditions that mimic the intracellular niche of the BCV (pH_4.4_; referred to here as the “low- pH” group) or normal-pH conditions (pH_7.3_; “normal-pH” group). The raw sequence outputs for each group are presented in Table 1. An MDS analysis revealed four clusters, of which all bacterial samples within each cluster are closely related, emphasizing the high quality and reproducibility of the data (Figure 1).

**Table 1:** The list of raw data sequence output for the normal- and low-pH groups of *B. melitensis* 16M and Rev.1.

Sample	Number of reads	Number of mapped reads	Number of unique reads	Number of multireads
c16M_1	10100002	9936571	9166680	769891
c16M_2	11159803	10971670	9999629	972041
c16M_3	11578986	11368597	10406648	961949
c16M_4	11983881	11809453	10675444	1134009
c16M_5	10342149	10171502	9243044	928458
e16M_1	12883209	11245980	10323376	922604
e16M_2	13370804	11211603	10325228	886375
e16M_3	13314490	10637845	9800648	837197
e16M_4	12717414	10407763	9768405	639358
e16M_5	12523271	10787022	10183357	603665
cRev1_1	9453360	9143362	8564979	578383
cRev1_2	12195263	11691352	10853987	837365
cRev1_3	13145448	12644912	11557656	1087256
cRev1_4	10815300	10207826	9459182	748644
cRev1_5	11052077	10371169	9553236	817933
eRev1_1	11005387	10756038	10093466	662572
eRev1_2	10892535	10654239	9949716	704523
eRev1_3	10208763	9979844	9441165	538679
eRev1_4	12700928	12464941	12176241	288700
eRev1_5	10150825	9873463	9665201	208262


*c: control (normal pH) group; e: experimental (low pH) group.*

Below, we first report the genes that are differentially expressed (DE) between the Rev.1 and 16M strains, each grown under normal-pH conditions. Then, for each separate strain, we report the genes that are DE between bacteria grown under normal-pH conditions and those grown under low-pH conditions. Finally, we report possible interactions between the strain and its unique response to acidic conditions.

### Differential Gene Expression Between *B. melitensis* Rev.1 and 16M Grown Under Normal-pH Conditions

**FIGURE 2:**
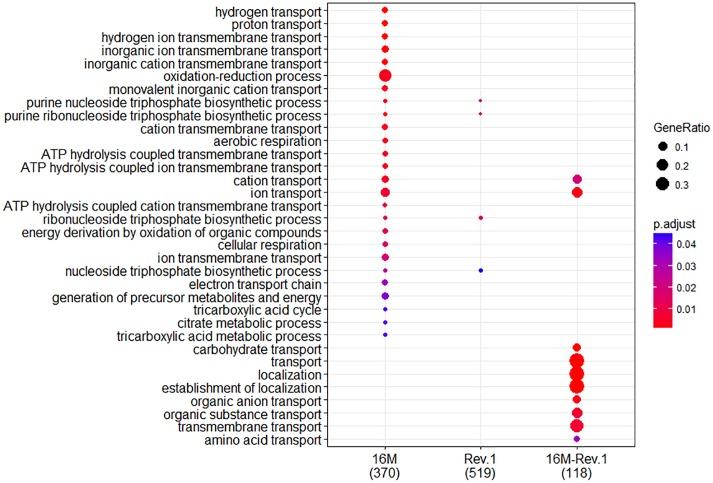
Comparison of biological Gene Ontology (GO) enrichment of the *B.* *melitensis* 16M (left column) and Rev.1 (middle column) genes that were differentially expressed when the bacteria were grown under low-pH versus normal-pH conditions. The right column (16M–Rev.1) indicates genes that were differentially expressed in the normal-pH Rev.1 group compared with the normal-pH 16M group. The dot-plot displays two layers of information: significance of enrichment (*p*-value), which is represented by the color of the dot (highly enriched: red; lowly enriched: blue), and the “gene ratio,” which is the degree of overlap between genes in the tested list and the genes associated with a GO term, represented by the size of each dot (large dots indicate a higher degree of overlap).

**FIGURE 3:**
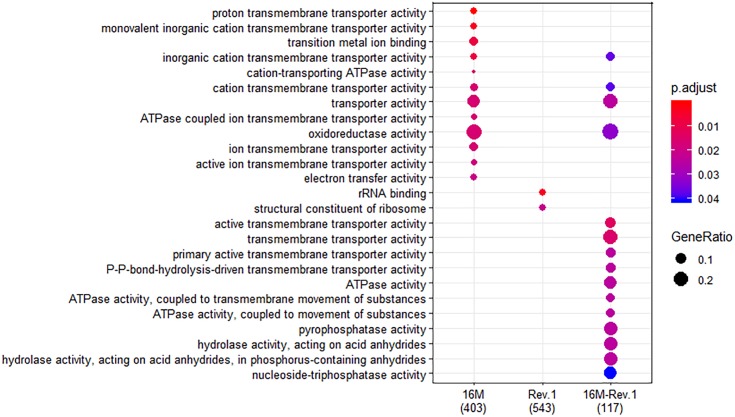
Comparison of molecular Gene Ontology (GO) enrichment of the *B.* *melitensis* 16M (left column) and Rev.1 (middle column) genes that were differentially expressed when the bacteria were grown under low- versus normal-pH conditions. The right column (16M–Rev.1) indicates genes that were differentially expressed in the normal-pH group of Rev.1, as compared with the normal-pH group of 16M. The dot-plot displays two layers of information: significance of enrichment (*p*-value), which is represented by the color of the dot (highly enriched: red; lowly enriched: blue), and the “gene ratio,” which is the degree of overlap between genes in the tested list and the genes associated with a GO term, represented by the size of each dot (large dots indicate a higher degree of overlap).

When both Rev.1 and 16M were grown under normal-pH conditions (pH_7.3_), our comparative transcriptomic analysis revealed 242 genes that were DE (FDR < 0.05, fold change ≥ 2; Supplementary Table S2) between the two strains, of which 172 genes were upregulated and 70 genes were downregulated in Rev.1 versus 16M. The most enriched biological processes associated with the DE genes were transport-related processes (Figure 2), while the most enriched molecular functions were cation transmembrane transporter, oxidoreductase, hydrolase, and ATPase activities (Figure 3). Twelve of the 242 DE genes encode for proteins that were previously reported in a proteomic analysis to be overexpressed in Rev.1 versus 16M (Table 2), including BMEII0704 (which encodes bacterioferritin) and six genes that encode ABC transporters (Forbes and Gros, 2001).

**Table 2:** List of DE genes found in the reported proteomic analysis.

Gene ID	Gene description	RNA-seq/proteome
BMEI0372	Response regulator	Overexpression/overexpression
BMEI1211	Amino acid ABC transporter substrate-binding protein	Overexpression/overexpression
BMEI1390	ABC transporter substrate-binding protein	Overexpression/overexpression
BMEI1923	Isovaleryl-CoA dehydrogenase	Overexpression/overexpression
BMEII0203	ABC transporter substrate-binding protein	Overexpression/overexpression
BMEII0344	ABC transporter permease	Overexpression/overexpression
BMEII0550	Glycine/betaine ABC transporter substrate-binding protein	Overexpression/overexpression
BMEII0590	Sugar-binding periplasmic protein	Overexpression/overexpression
BMEII0633	Branched-chain amino acid ABC transporter substrate-binding protein	Overexpression/overexpression
BMEII0704	Bacterioferritin	Overexpression/overexpression
BMEII0746	2-Oxo acid dehydrogenase subunit E2	Overexpression/overexpression
BMEII0747	Alpha-ketoacid dehydrogenase subunit beta	Overexpression/overexpression


Next, we compared the genes that we found to be DE between the two strains to a list of *Brucella* virulence genes obtained from the *Brucella* Bioinformatics Portal (Xiang et al., 2006). Out of the 212 *B. melitensis* virulence genes that were reported in the *Brucella* Bioinformatics Portal, our transcriptomic analysis indicated eight genes that were upregulated and eight genes that were downregulated in Rev.1 versus 16M (Table 3), including six genes that encode transporters, of which three are annotated as sugar transporters.

**Table 3:** List of DE genes found in the *Brucella* Bioinformatics Portal.

Gene ID	Gene description	Up/downregulation (Rev.1 versus 16M)
BMEI0451	2-Isopropylmalate synthase	Upregulation
BMEI0545	DUF475 domain-containing protein	Downregulation
BMEI0624	Ketol-acid reductoisomerase	Downregulation
BMEI0626	PLP-dependent aminotransferase family protein	Upregulation
BMEI0796	Hypothetical protein	Downregulation
BMEI1759	Methionine synthase	Downregulation
BMEI1837	DUF3131 domain-containing protein	Downregulation
BMEII0056	Magnesium-translocating P-type ATPase	Upregulation
BMEII0077	Isochorismate synthase	Upregulation
BMEII0361	Sugar ABC transporter ATP-binding protein	Upregulation
BMEII0591	Sugar ABC transporter permease	Upregulation
BMEII0761	Thiol reductant ABC exporter subunit CydC	Downregulation
BMEII0762	Thiol reductant ABC exporter subunit CydD	Downregulation
BMEII0923	ABC transporter substrate-binding protein	Upregulation
BMEII0931	Class Ib ribonucleoside-diphosphate reductase assembly flavoprotein NrdI	Downregulation
BMEII1053	glucose/galactose transporter	Upregulation


### Differential Gene Expression Between *B. melitensis* 16M Grown Under Low- and Normal-pH Conditions

In total, 773 genes in the 16M strain were DE (FDR < 0.05, fold change ≥ 2) between bacteria grown under normal- and low-pH conditions, of which 374 were upregulated and 399 were downregulated in the low-pH group versus the normal-pH group (Supplementary Table S3). The most enriched biological processes within these DE genes were transport, oxidation–reduction, and nucleoside triphosphate biosynthetic processes (Figure 2), and the most enriched molecular functions were ion and cation transmembrane transporters, oxidoreductase activities, and transition metal ion binding (Figure 3). Recently, Liu et al. (2016) reported 113 genes that are DE (using FDR < 0.05 and fold change ≥ 8) between normal- and low-pH conditions in 16M. Of these genes, 104 were also annotated in our analysis, of which 72 were DE (∼70%; FDR < 0.05, fold change ≥ 2; Supplementary Table S4) between the two conditions, including 24 genes that were upregulated and 48 genes that were downregulated in the low-pH group versus the normal-pH group. Notably, the two-component response regulator BMEI1329, which is involved in the acid resistance of *B. melitensis* (Liu et al., 2016) was upregulated to a similar extent (namely, a fold-change of ∼4.55) in our analysis and in that of Liu et al. (2016).

### Differential Gene Expression Between *B. melitensis* Rev.1 Grown Under Normal- and Low-pH Conditions

In total, 1076 genes in the Rev.1 strain were DE (FDR < 0.05, fold change ≥ 2) between the low-pH and normal-pH groups, of which 519 genes were upregulated and 557 genes were downregulated in the low-pH versus the normal-pH group (Supplementary Table S5). The most enriched biological process within these DE genes was the nucleoside triphosphate biosynthetic process (Figure 2), and the most enriched molecular functions were rRNA binding and a structural constituent of the ribosome (Figure 3).

### The Effects of Low-pH Conditions on Gene Expression in *B. melitensis* 16M and Rev.1: A Comparison

**FIGURE 4:**
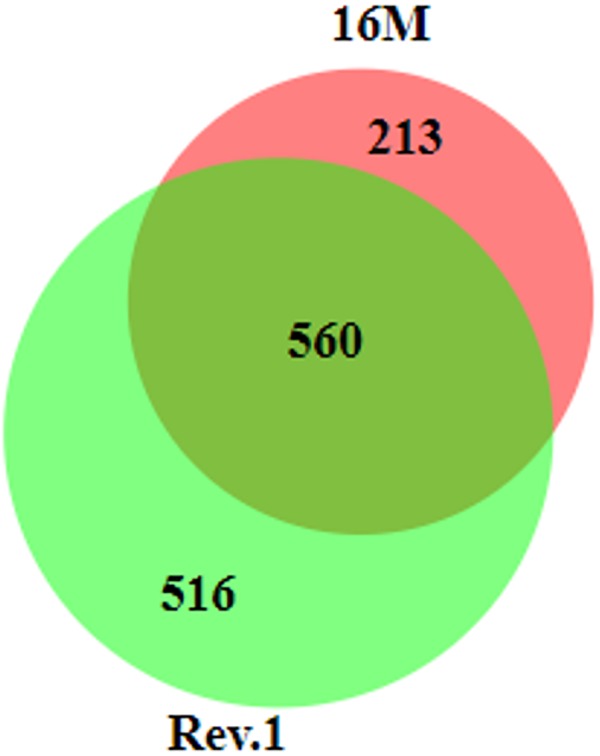
Venn diagram of differentially expressed genes (fold change ≥ 2 and FDR < 0.05) in 16M and Rev.1 under low-pH conditions. The size of the circles and the overlaps correspond to the sizes of the data sets (number of DE genes in each strain). The numbers of overlapping and unique genes in each strain are indicated in the plot. The diagram was generated using BioVenn (Hulsen et al., 2008).

In total, 560 genes that were DE between the low-pH and normal-pH conditions were common to both 16M and Rev.1, while 213 and 516 of the DE genes were unique to either 16M or Rev.1, respectively (Figure 4). A comparison of the genes that uniquely changed their expression between the low-pH and normal-pH groups in Rev.1 to those that uniquely changed their expression between the two conditions in 16M, in relation to their GO categories, revealed that the main biological processes that were highly enriched in Rev.1 were translation, metabolic process, and transport (transmembrane, amino acid, carbohydrate, and protein), whereas multiple biological processes were enriched in 16M, including pathogenesis, cell division, cell cycle, and cell wall organization (Supplementary Table S6).

### Interaction Genes: Determination of a Possible Link Between Gene Expression, Environmental Stress, and a Specific Strain

**FIGURE 5:**
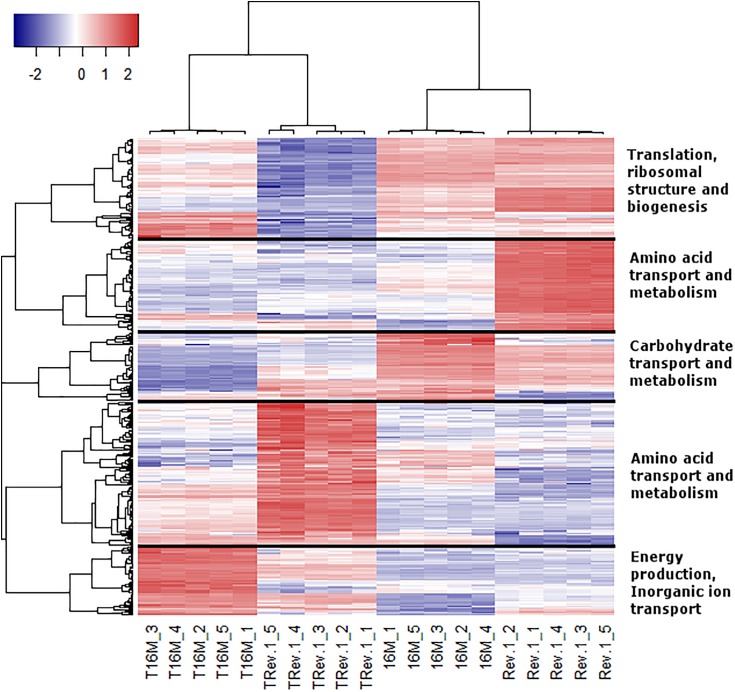
Heatmap representing the expression profiles of the 403 interaction genes. Rows represent genes and columns represent bacterial samples. Red and blue pixels indicate upregulated and downregulated genes (Rev.1 versus 16M), respectively. The hierarchical clustering was generated using 1-Pearson correlation as the distance measure, and “complete” as the linkage method. Genes were categorized into five clusters based on the generated dendrogram, and genes within each cluster were characterized based on Clusters of Orthologous Groups (COGs) annotations.

**FIGURE 6:**
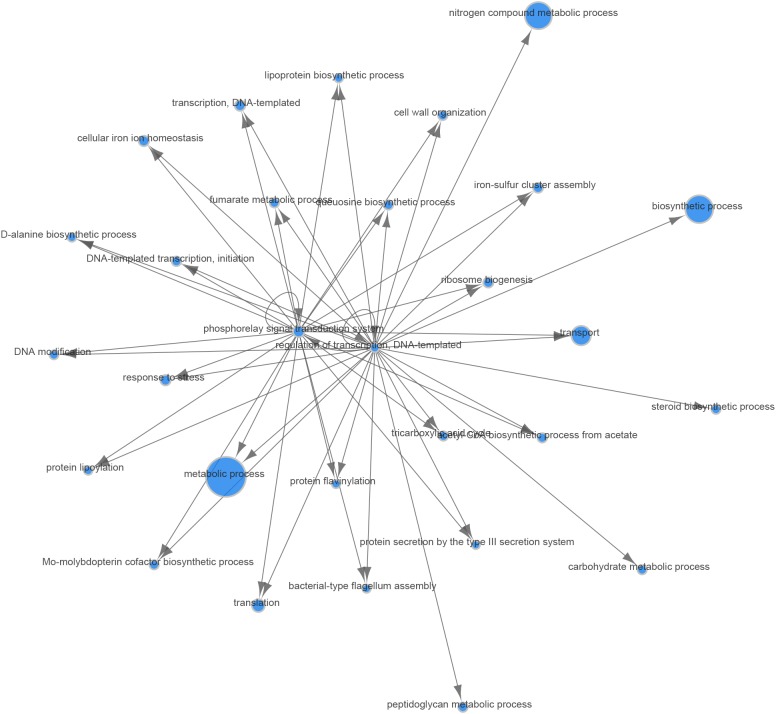
Gene Ontology (GO) network of genes responding differently to low-pH conditions in the Rev.1 versus 16M strains. Of the 403 interaction genes (FDR < 0.05, fold change ≥ 2) expressed differently in Rev.1 versus 16M, the 133 most significant genes (FDR < 0.05, fold change ≥ 2.8) were selected and their GO network was generated using the Comparative GO tool (Fruzangohar et al., 2013). The node sizes represent the level of GO enrichment.

In the analyses described above, we assumed that the two major parameters that could affect gene expression—the specific *B. melitensis* strain (Rev.1 versus 16M) and the environmental pH (4.4 versus 7.3)—are independent. Therefore, we adopted a naive approach and detected the effect of the acidic environment on gene expression in each strain separately, then compared the final list of DE genes. Our next step was to identify the potential dependency between strain and environmental pH, i.e., we sought to detect genes that respond differently to acidic stress in Rev.1 versus 16M. To this end, we added the “interaction” term to our statistical model, which revealed 403 genes that can be referred to as “interaction genes” (FDR < 0.05, fold change ≥ 2; Supplementary Table S7) and may potentially shed light on the attenuation mechanisms of Rev.1. Annotating these “interaction genes” revealed that the most enriched biological processes were related to metabolic, biosynthetic, and transport processes; the most enriched molecular functions were related to catalytic, hydrolase, nucleotide binding, oxidoreductase, and transporter activities; and the most enriched cellular compartments were related to integral components of the membrane (Supplementary Tables S8–S10). To identify genes that are potentially involved in the attenuation and survival of Rev.1, we searched the interaction genes for those that are associated with bacterial virulence and survival within the host and found four highly downregulated genes involved in metabolism processes and mitigation of acidic and oxidative stresses (Table 4). A heatmap of the 403 detected interaction genes, categorized into five clusters, is presented in Figure 5, and the list of genes within each cluster is shown in Supplementary Table S11. Finally, we created a GO Network based on 133 interaction genes with FDR < 0.05 and fold change ≥ 2.8 (Figure 6); as expected, the most enriched GO was related to transport and metabolic processes.

**Table 4:** Potentially key genes involved in the attenuation of Rev.1.

Gene ID	Gene	Fold change
BMEII0815	Acetyl-coenzyme A (acetyl-CoA) synthetase	16.9
BMEI1747	Aldehyde dehydrogenase	5.06
BMEI1980	DNA starvation/stationary phase protection protein Dps	3.3
BME_RS02600	Cold-shock protein	2.19


### RT-qPCR Validation of the RNA-Seq Results

To ensure technical reproducibility and to validate the data generated from the RNA-seq experiment, we conducted a real-time qPCR analysis of five selected genes (BMEII0027, BMEII0591, BMEI1980, BMEII1116, and BMEI1040), from both strains (16M and Rev.1), grown under either low- or normal-pH conditions. The mRNA levels of all genes obtained by the RT-qPCR were in high accordance with those obtained by our RNA-seq analysis (Supplementary Table S1).

### Differential Survival of *B. melitensis* 16M and Rev.1 Within JEG-3 Human Trophoblastic Cells

**FIGURE 7:**
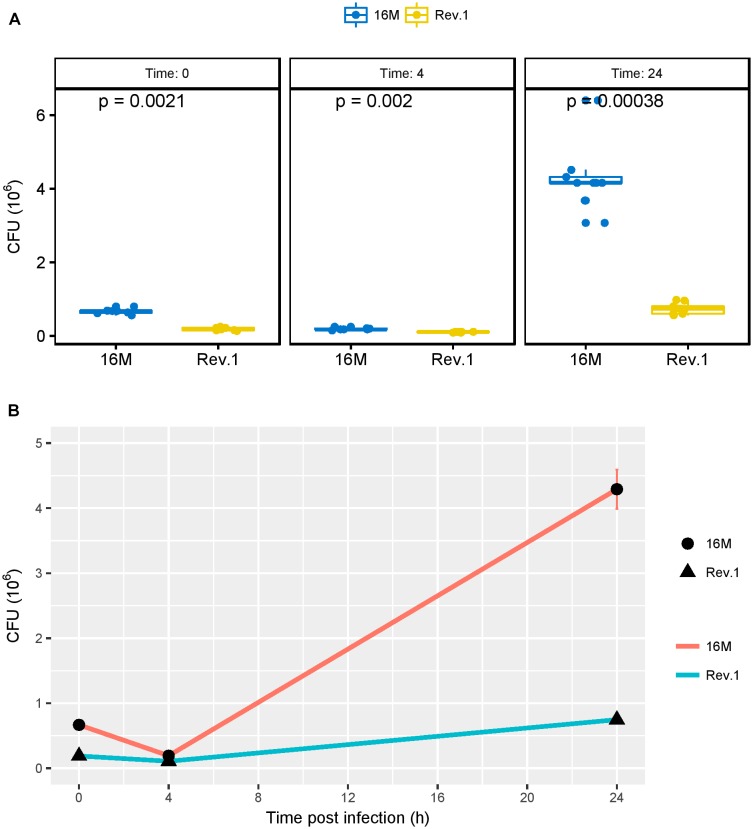
Bacterial burden, measured as colony forming units (CFUs) over time. JEG-3 trophoblasts were infected with bacteria at a MOI of 500, and CFUs were determined 0, 4, and 24 h thereafter. **(A)** Boxplots representing the distribution of 16M (blue) and Rev.1 (yellow) bacterial count replicates, measured 0, 4, and 24 h following infection. *P-*values were calculated using two-sample Wilcoxon test. **(B)** Scatter plot indicating the change in bacterial counts over time (means ± SD) following infection. Three replicate wells were evaluated at each time for each strain.

As shown by Porte et al. (1999), the acidic environment during the early phase of infection is necessary for the survival and multiplication of *Brucella* in host cells. Therefore, we investigated the ability of the virulent 16M and the attenuated Rev.1 strains to infect and replicate within the human trophoblastic cell line JEG-3. As expected, the number of bacteria that were replicated over time (4 and 24 h) was higher in 16M than in Rev.1 (Figure 7).

## Discussion

To elucidate the molecular mechanisms underlying the attenuation of the *B. melitensis* Rev.1 vaccine strain, we conducted a comparative transcriptomic analysis between Rev.1 and its virulent counterpart, 16M, each grown under either normal- or low-pH conditions. When the two strains were grown under normal-pH conditions, Rev.1 showed a marked upregulation, as compared with 16M, of various genes that encode ABC transporters—a large and widespread family of proteins (Garmory and Titball, 2004). ABC transporters, which export solutes, antibiotics, and extracellular toxins, play a role in various cellular processes, such as translational regulation and DNA repair (Garmory and Titball, 2004), and the number of ABC systems appears to depend upon the bacterial adaptation to its environment (Garmory and Titball, 2004; Tanaka et al., 2018). The upregulation of ABC transporter-related genes in the attenuated Rev.1 strain should be considered in light of two other findings. First, Rev.1 showed an upregulated expression of BMEII0704, which encodes for bacterioferritin (Table 2). Notably, iron plays an important role in the survival of pathogens within host cells (Collins, 2003), and during infection, macrophages actively export iron from the phagosome (which is the replicative niche of *Brucella*; Forbes and Gros, 2001); it was previously suggested that Rev.1 may have lost the ability to regulate bacterioferritin synthesis and degradation (Eschenbrenner et al., 2002). Second, Rev.1 showed a downregulated expression of BMEI1759 (Table 3), which encodes for the vitamin B12-dependent methyltransferase, MetH (Lestrate et al., 2000). As MetH is involved in methionine biosynthesis, its downregulation in Rev.1 may have impaired amino acid metabolism in this strain. Taken together, these finding suggest that an improper regulation of essential metabolic pathways, including iron and amino acid metabolism, may have affected the ABC transporter activity, leading to down/upregulation of specific transporters to compensate for the gain/loss of critical metabolites.

To further understand the molecular mechanisms underlying Rev.1 attenuation, we examined the “interaction genes,” i.e., the main genes that are influenced differently by the acidic treatment in Rev.1 and in 16M. These interaction genes are probably the key genes involved in the attenuation of Rev.1, and are, therefore, of particular interest. Among these interaction genes, we found that acetyl-coenzyme A (acetyl-CoA) synthetase was significantly downregulated in the low-pH group of Rev.1, as compared with the low-pH group of 16M. Acetyl-CoA is the molecule by which glycolytic pyruvate enters the tricarboxylic acid cycle, it is a crucial precursor of lipid synthesis, and it acts as a sole donor of the acetyl groups for acetylation (Pietrocola et al., 2015). Previous studies revealed a clear association between the metabolism of *Brucella* and its persistence in its hosts (Hong et al., 2000; Lestrate et al., 2000; Barbier et al., 2011). The significantly high downregulation of acetyl-CoA synthetase in Rev.1 may decrease the levels of acetyl-CoA production, thereby affecting crucial metabolic processes that may potentially have a major contribution to bacterial attenuation.

Pathogenic bacteria must deal with oxidative stress emanating from the host immune response during invasion and persistent infection (Cabiscol et al., 2000; Singh et al., 2013). We found several key interaction genes that were significantly downregulated in Rev.1, as compared with 16M, and which encode proteins involved in oxidative stress: aldehyde dehydrogenase (ALDH), the DNA starvation/stationary phase protection protein Dps, and a cold-shock protein (CSP). Prokaryotic and eukaryotic ALDHs metabolize endogenous and exogenous aldehydes to mitigate oxidative stress, (Singh et al., 2013) and an upregulation of bacterial ALDH was shown to occur following exposure to environmental or chemical stressors. It was suggested that such an upregulation is a critical element in the response of bacteria to oxidative stress (Singh et al., 2013). Dps was shown to protect *Escherichia coli* from oxidative stress, UV and gamma irradiation, iron and copper toxicity, thermal stress, and acid and base shocks (Martinez and Kolter, 1997; Nair and Finkel, 2004; Karas et al., 2015). CSP-A activity was found to be associated with the ability of *B. melitensis* to resist acidic and H_2_O_2_ stresses, especially during the mid-log-phase (Wang et al., 2014), and it was suggested that the *Brucella* CSP highly contributes to its virulence, most likely by facilitating its adaptation to the harsh environmental circumstances within the host (Wang et al., 2014). Taken together, it is possible that the downregulation of these key interaction genes in Rev.1 results in its inability to cope with oxidative stress in the host, thus contributing to bacterial attenuation.

As compared with Rev.1, 16M demonstrated a downregulation (FDR < 0.05, fold change ≥ 2) of interaction genes that encode for four proteins of the SUF system and the heat-shock protein IbpA, which were shown to be involved in the resistance to heat and oxidative stress (Kitagawa et al., 2000; Angelini et al., 2008; Outten, 2015). In *E. coli*, the SUF pathway plays a major role in preserving Fe-S cluster biosynthesis under oxidative stress conditions (Angelini et al., 2008; Outten, 2015). The small heat shock proteins (sHsps) IbpA and IbpB were previously suggested to be involved in the resistances to heat and oxidative stress, as overexpression of *ibpA* and *ibpB* in *E. coli* increased the resistance to heat and superoxide stress (Kitagawa et al., 2000). Our transcriptomic analysis revealed enhanced enrichment within the molecular function of oxidoreductase activity in the low-pH group of 16M, but not in the low-pH group of Rev.1. As oxidoreductase protects bacteria against oxidative stress (Lumppio et al., 2001), we assume that the enhanced expression of the SUF system and IbpA by Rev.1, as compared with 16M, compensates for its impaired oxidoreductase activity, thereby enabling survival within the harsh oxidative intracellular environment of the host.

As compared with the low-pH group of 16M, the low-pH group of Rev.1 showed a downregulation (FDR < 0.05, fold change ∼ 2) of five key interaction genes BME_RS02910, BMEI0584, BMEI0583, BME_RS13825, and BMEI1943 that encode for FtsZ, FtsA, FtsQ, FtsK, and DnaA respectively, all of which participate in critical stages of the cell cycle (Margolin, 2005; Sherratt et al., 2010; van den Ent et al., 2008; Bell and Kaguni, 2013; Loose and Mitchison, 2014). This finding may indicate that, in the acidic conditions of the BCV, the replication capabilities of Rev.1 are reduced, which lowers their intracellular survival compared to 16M. The significantly lower intracellular survival of Rev.1 in trophoblasts (Figure 7) supports this conclusion.

Under low-pH conditions, Rev.1 also showed a downregulation of the interaction gene BMEII1048 that encodes the molecular chaperone, GroEL (FDR < 0.05, fold change ∼ 2). Molecular chaperons facilitate protein folding, preventing protein denaturation, and are involved in various cellular processes, including DNA replication, UV mutagenesis, bacterial growth, and RNA transcription (Maleki et al., 2016). Under acidic conditions, partially unfolded proteins may emerge (Mendoza et al., 2017) and molecular chaperones may stabilize them to prevent their acid-induced aggregation. Indeed, the *Helicobacter pylori* GroEL homolog, HSP60, was shown to be induced upon acid stress (Mendoza et al., 2017). Thus, the downregulation of the interaction gene encoding GroEL in Rev.1 under low-pH conditions may lead to the accumulation of partially unfolded and desaturated proteins, leading to bacterial attenuation.

Finally, the interaction gene BMEII0027, which encodes for the T4SS protein VirB3, was upregulated (FDR < 0.05, fold change ∼ 2) in the low-pH group of 16M, as compared with the low-pH group of Rev.1. This may have a harmful effect on the survival of Rev.1 within host cells, as VirB3 was shown to be essential for *Brucella* virulence because, together with VirB4, VirB6, VirB8, and the N-terminus of VirB10, it comprises the inner membrane complex of the T4SS apparatus (Ke et al., 2015).

Notably, the Rev.1 upregulated interaction gene BMEII1116 that encodes the HTH-type quorum sensing-dependent transcriptional regulator VjbR, was shown to contribute to the virulence and survival of *Brucella* by regulating the expression of various virulence factors (Delrue et al., 2005; Weeks et al., 2010). It is possible that this interaction gene somewhat compensates for the lower expression of highly important virulent genes, such as VirB3, in Rev.1.

## Conclusion

Through a comparative transcriptomic analysis, we revealed DE key genes involved in various crucial pathways, which are either upregulated or downregulated under acidic conditions in Rev.1, as compared with 16M. We suggest that these genes—and, especially, those mentioned in Table 4—are involved in the molecular mechanisms underlying Rev.1 attenuation, although further characterization through mutation and knockout experiments is required to conclusively determine the role of these genes in acid resistance and virulence attenuation of the *B. melitensis* Rev.1 strain.

## Author Contributions

MS-D and DK conceived and coordinated the study. DK conducted the bacteriology work, acidic experiments, acquired the samples, and extracted RNA. MS-D analyzed the data. TZ performed the real-time PCR validation experiments. All authors interpreted the data, drafted the manuscript, and approved the content for publication.

## Conflict of Interest Statement

The authors declare that the research was conducted in the absence of any commercial or financial relationships that could be construed as a potential conflict of interest.

## Footnotes

1 http://www.bioinformatics.babraham.ac.uk/projects/fastqc

2 http://www.bioinformatics.babraham.ac.uk/projects/trim_galore/

3 https://github.com/aleimba/bac-genomics-scripts/tree/master/cdd2cog
